# Photocatalytic and Adsorption Performances of Faceted Cuprous Oxide (Cu_2_O) Particles for the Removal of Methyl Orange (MO) from Aqueous Media

**DOI:** 10.3390/molecules22040677

**Published:** 2017-04-23

**Authors:** Weng Chye Jeffrey Ho, Qiuling Tay, Huan Qi, Zhaohong Huang, Jiao Li, Zhong Chen

**Affiliations:** 1 School of Materials Science and Engineering, Nanyang Technological University, Singapore 639798, Singapore; qltay@ntu.edu.sg (Q.T.); QIHU0002@ntu.edu.sg (H.Q.); 2 Singapore Institute of Manufacturing Technology, 71 Nanyang Drive, Singapore 638075, Singapore; zhhuang@SIMTech.a-star.edu.sg; 3 School of Materials Science and Engineering, Shandong University of Technology, Zibo 255049, Shandong Province, China; haiyan9943@163.com

**Keywords:** cuprous oxide (Cu_2_O), adsorption, photocatalytic performance, methyl orange (MO), dye degradation, photocorrosion

## Abstract

Particles of sub-micron size possess significant capacity to adsorb organic molecules from aqueous media. Semiconductor photocatalysts in particle form could potentially be utilized for dye removal through either physical adsorption or photo-induced chemical process. The photocatalytic and adsorption capabilities of Cu_2_O particles with various exposed crystal facets have been studied through separate adsorption capacity test and photocatalytic degradation test. These crystals display unique cubic, octahedral, rhombic dodecahedral, and truncated polyhedral shapes due to specifically exposed crystal facet(s). For comparison, Cu_2_O particles with no clear exposed facets were also prepared. The current work confirms that the surface charge critically affects the adsorption performance of the synthesized Cu_2_O particles. The octahedral shaped Cu_2_O particles, with exposed {111} facets, possess the best adsorption capability of methyl orange (MO) dye due to the strongest positive surface charge among the different types of particles. In addition, we also found that the adsorption of MO follows the Langmuir monolayer mechanism. The octahedral particles also performed the best in photocatalytic dye degradation of MO under visible light irradiation because of the assistance from dye absorption. On top of the photocatalytic study, the stability of these Cu_2_O particles during the photocatalytic processes was also investigated. Cu(OH)_2_ and CuO are the likely corrosion products found on the particle surface after the photocorrosion in MO solution. By adding hole scavengers in the solution, the photocorrosion of Cu_2_O was greatly reduced. This observation confirms that the photocatalytically generated holes were responsible for the photocorrosion of Cu_2_O.

## 1. Introduction

One of the main issues that human society faces in the 21st century is pollution of water and air caused by the increasing industrial activities and human consumption of natural resources including minerals, coal, petroleum and their derivatives. Pollution poses serious threat to not only human health, but also marine life, which in turns affects livelihood of human being through the food chains. Membrane technology provides an effective way to extract clean water from polluted water, however the equipment and processing cost is high, as the technology requires a large amount of electricity and space. Alternative cost-effective approaches are needed to not only remove, but also, ideally, chemically decompose the organic pollutants in wastewater. Semiconductor photocatalysts offer such a possibility.

Since the discovery of hydrogen production through photocatalytic reactions by Fujishima and Honda [1] in 1972, enormous efforts have been devoted to its development [2,3]. Besides hydrogen evolution, photocatalysts are also capable of purifying wastewater, simply by breaking down the organic molecules using chemical reactions energized by the renewable solar light [4,5,6]. Devising suitable semiconductor photocatalysts for the decomposition of organic compounds using solar energy has been a major topic in the research community in recent decades. Substantial progress has been made, and some of the recent development has been summarized in [7,8,9,10,11,12].

Nanoparticulated photocatalysts offer clear advantages in this regard, as they possess a larger specific surface area for photo absorption and more active sites for the catalytic reactions [13]. If the surface of these particles is properly engineered, these particles can also physically adsorb the pollutant molecules from wastewater [14]. Therefore, semiconductors in particle form can be potentially applied to remove organic pollutants from aqueous solution through either chemical (photocatalytic) reactions, or physical adsorption, or both. It has been demonstrated that photocatalysis and adsorption can be applied synergistically to speed up the removal of pollutants from aqueous media [4,6,14]. This also implies that, when assessing the photocatalytic activity based on pollutant removal from the aqueous solution (typically by measuring the waste molecule concentration in water), care has to be taken to evaluate the effectiveness from photocatalytic action and adsorption separately.

Cuprous oxide (Cu_2_O), due to its relatively narrow band gap among semiconductor metal oxides, has received lots of attention in recent years as visible light photocatalyst [15,16,17,18]. Particularly, faceted single crystal Cu_2_O particles have been synthesized from Cu salts using different type of reducing agents. Their surface, electrical and catalytic properties have been studied. It has been acknowledged that particles with different exposed facets possess different physical and chemical properties, thus the simple solution process for crystal facet-control provides an attractive means to improve the material’s properties. However, thus far the reported photocatalytic activity among different Cu_2_O facets differs depending on the type of organic pollutants used as well as the experimental conditions. Huang et al. [15] prepared cubic, cuboctahedra, truncated octahedral, octahedral, and multipod structured Cu_2_O nanocrystals and compared their photocatalytic activities. They found that octahedra and hexapods with the {111} exposed facets are catalytically most active in photocatalytically decomposing negatively charged molecules such as methyl orange (MO), while cubes with only the {100} faces are not photocatalytically active. In one of the following papers from the same group [16], rhombic dodecahedra exposing only the {110} facets were reported to exhibit the best photocatalytic activity among cubic, face-raised cubic, edge- and corner-truncated octahedral, all-corner-truncated rhombic dodecahedral, {100}-truncated rhombic dodecahedral, and rhombic dodecahedral Cu_2_O particles. In both cases, the authors attributed the activity to the high number of surface copper atoms on the exposed facets [15,16].

When nano or sub-micron sized particles are used, it is important to study both adsorption and photocatalytic performances in order to reach a comprehensive conclusion. In the current work, we also chose the commonly used organic dye, MO, as the model pollutant. Cu_2_O particles with different types of exposed facets were synthesized and compared for their adsorption and visible light photocatalytic activities. Since Cu_2_O is well known for its instability under photocatalytic testing condition [19], comparison was also made on the photo stability of these particles. The mechanism for the photocorrosion was investigated based on corrosion product analysis.

## 2. Results and Discussion

### 2.1. Morphology and Crystal Structure of the Synthesized Cu_2_O and Surface Area

**Figure 1:**
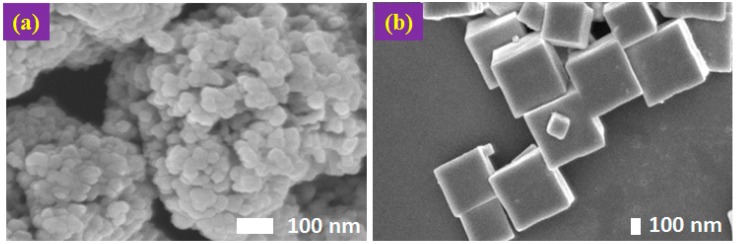
Field Emission Scanning Electron Microscopy (FESEM) images of Cu_2_O particles: (**a**) Cu_2_O of agglomerated spherical shape with no clear exposed facets; and (**b**) Cu_2_O of cubic shape with exposed {100} facets.

The average size of the Cu_2_O particles with standard deviation is summarized in Table 1. When 0.01 M of NaOH was used, porous Cu_2_O particles of about 0.8 μm in size were obtained (Figure 1a). The particles consisted of agglomerated (non-separable) smaller nanoparticles with mean size around 40 nm. The small nanoparticles have no specific exposed facets, and the agglomerated cluster has an equal axial shape. For convenience, this morphology is denoted as spherical Cu_2_O particles. When NaOH concentration increased from 0.01 to 0.03 M, the particle was transformed into cubic shape with six {100} facets exposed (Figure 1b). To form the cubic shape, the growth rate in the <100> direction must be the slowest among all other growth directions. This was achieved by an increased NaOH concentration together with the addition of ascorbic acid. The obtained cubic Cu_2_O particles show uniform size and sharp edges.

**Figure 2:**
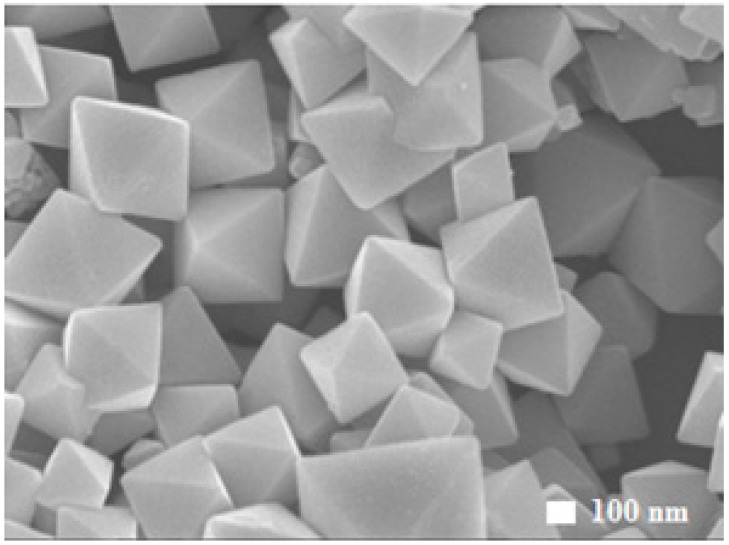
FESEM images of octahedral shaped Cu_2_O particles exposing the {111} facets.

Figure 2 shows the Field Emission Scanning Electron Microscopy (FESEM) image of well-defined, smooth and uniform octahedral shaped Cu_2_O particles. Each particle has eight exposed {111} facets. The synthesis method was similar to the formation of the cubic shape, except that ascorbic acid was replaced by a stronger reducing agent, hydrazine hydrate.

**Figure 3:**
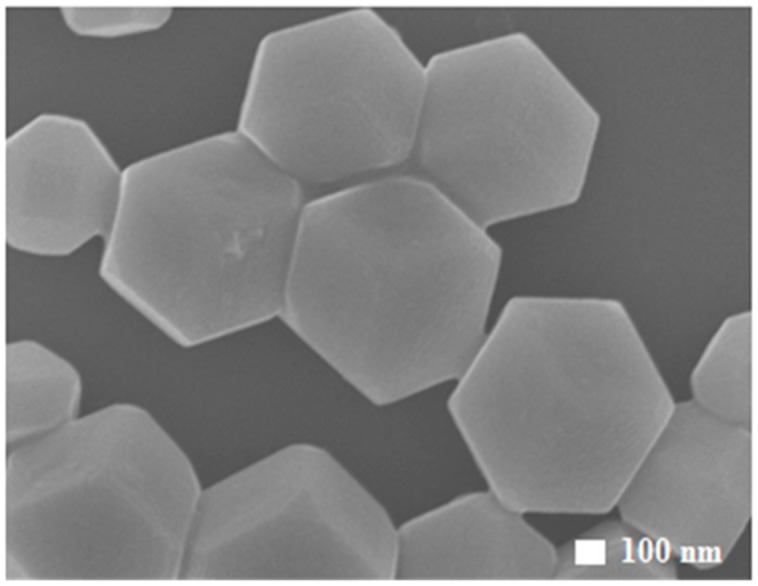
FESEM images of particles in rhombic dodecahedral shape with exposed {110} facets.

Figure 3 shows flat and smooth surfaces of rhombic dodecahedral Cu_2_O with 12 {110} facets. Both oleic acid and glucose were used in the synthesis. Apparently, they are able to reduce the growth along the <110> direction, resulting the exposed facets.

**Figure 4:**
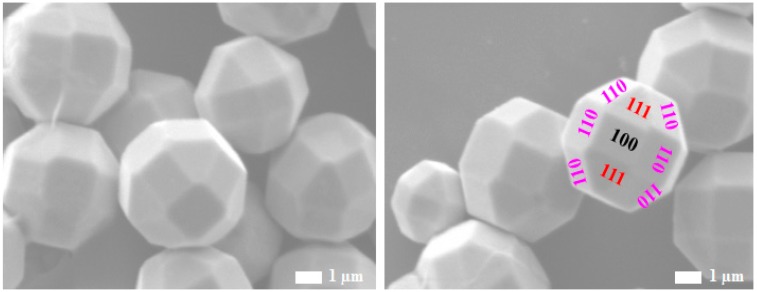
FESEM images of the truncated polyhedral Cu_2_O particles, exposing eight {111} facets, six {100} facets and 12 {110} facets.

Figure 4 shows the truncated polyhedral Cu_2_O particles with 26 facets. Among them, eight are the {111} facets (indicated in red) and six are the {100} facets (indicated in black). The remaining 12 are the {110} facets (indicated in pink). Due to the high concentration of the precursors used and longer cooling time (1.5 h), the size of the smooth and uniform truncated polyhedral Cu_2_O particle was seen to be relatively large (5 μm) compared to the other particles. It was observed that by lowering the concentration of the precursors and shortening the cooling time, some irregular cubic shaped Cu_2_O particles were obtained with an average size of 2 μm (not shown here). Therefore, both factors, the concentration and the cooling rate, are important to synthesize the truncated polyhedral Cu_2_O particles.

**Figure 5:**
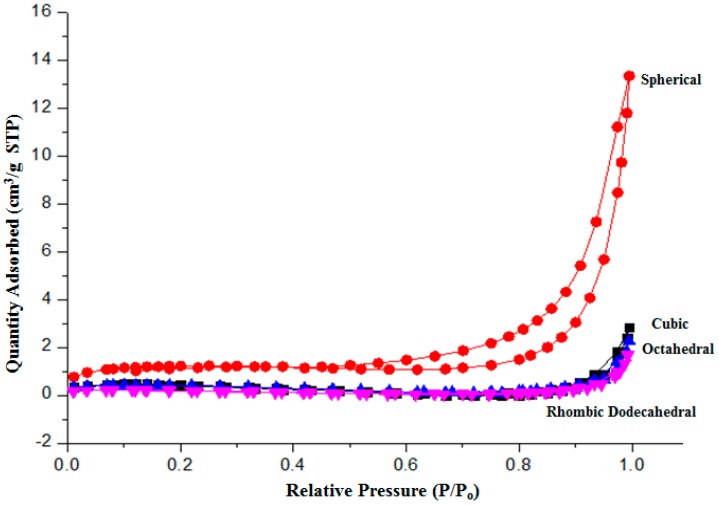
Isotherm N_2_ adsorption of Cu_2_O particles. STP: Standard Temperature and Pressure.

Figure 5 shows the nitrogen adsorption curves for the Cu_2_O samples. The Brunauer–Emmett–Teller (BET) surface area is summarized in Table 1. Not surprisingly, the spherical shaped Cu_2_O yields the highest surface area. The one for the truncated polyhedral Cu_2_O could not be measured, due to the large size of the particles exceeding the detection limit of the equipment (~2 μm). 

**Figure 6:**
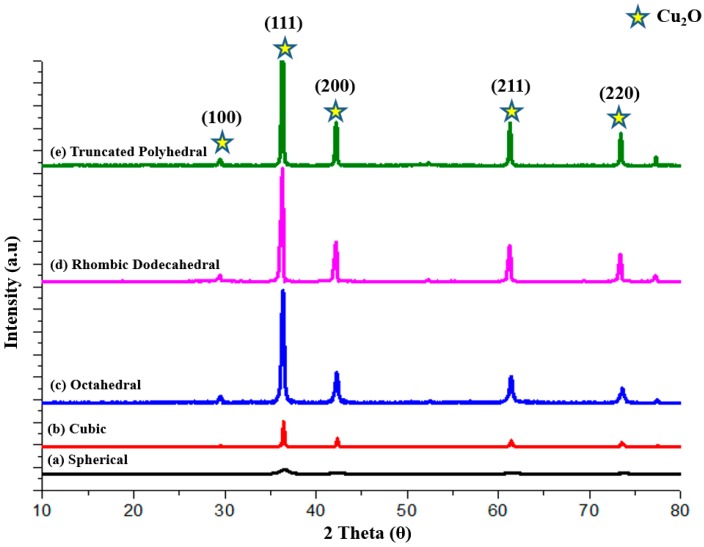
The X-ray diffraction (XRD) patterns of Cu_2_O particles: (**a**) spherical; (**b**) cubic; (**c**) octahedral; (**d**) rhombic dodecahedral; and (**e**) truncated polyhedral.

The crystal structure of various shaped Cu_2_O synthesized by the wet chemical method is confirmed by the X-ray diffraction (XRD) pattern shown in Figure 6. The diffraction peaks labeled with “★” matches well with the standard data of Cu_2_O (JCPDS No. 05-0667), confirming the formation of a single cubic phase structure. All the peaks were perfectly indexed to crystalline Cu_2_O not only in their peak positions (2θ values of 29.60°, 36.52°, 42.44°, 61.40° and 73.55°), but also in their relative intensity. It was observed that the XRD peaks for all the faceted samples were relatively sharp, due to the good crystallinity of the crystals. However, the crystallinity for the spherical shaped Cu_2_O particles was poor, as indicated by the weak and broad peaks.

### 2.2. FTIR and Zeta Potential Analyses

**Figure 7:**
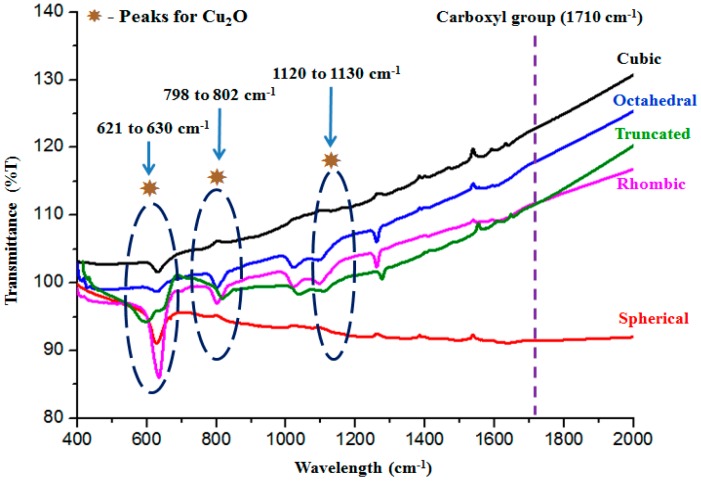
The Fourier Transform Infrared (FTIR) spectra of the faceted Cu_2_O particles.

The Fourier Transform Infrared (FTIR) spectra for all the five samples synthesized are shown in Figure 7. Though the spectrum was run from 400 to 4000 cm^−1^, the bands from metal oxide are generally below 1000 cm^−1^ [20]. For Cu_2_O, the bands that lie at 1130, 798 and 621 cm^−1^ were attributed to the stretching vibration of Cu–O in Cu_2_O. Thus, the results obtained have proven the existence of Cu_2_O. No bands related to CuO, which would appear at 588, 534 and 480 cm^−1^ [21], were detected. The use of oleic acid may lead to carboxylate (C=O stretch) on the surface of the particles which would show in the FTIR spectra at 1710 cm^−1^. Based on Figure 7, the peak for the carboxylate could not be detected across all the synthesized Cu_2_O particles. This clearly proves that the surfaces of the Cu_2_O particles are ligand free.

The zeta potentials for all the Cu_2_O samples were measured and the results are tabulated in Table 2. The surface charge of the spherical, cubic and octahedral shaped Cu_2_O was positively charged (indicated by the “+” sign) while the surfaces of the rhombic dodecahedral and truncated polyhedral Cu_2_O were negatively charged (indicated by the “−” sign). The surface charge is related to the surface bonding state on the exposed facets. Our observation that the octahedral particles with exposed {111} planes possess the most positive surface charge indicates that there are probably more un-coordinated Cu bonds on the particle surfaces. On the other hand, the surface charge of the rhombic dodecahedral Cu_2_O particles consisting of 12 {110} planes was found to be negative, suggesting the O ions are likely to be dominant on the exposed surface. This result is contrary to some previous reports [16,18] that claim the rhombic dodecahedral particles are positively charged based on visualizing the atomic models of Cu_2_O. Strictly speaking, this approach (cutting and visualizing a particular crystal model) cannot deduce the information on particle surface charge.

### 2.3. The Adsorption Performance of Cu_2_O Particles

**Figure 8:**
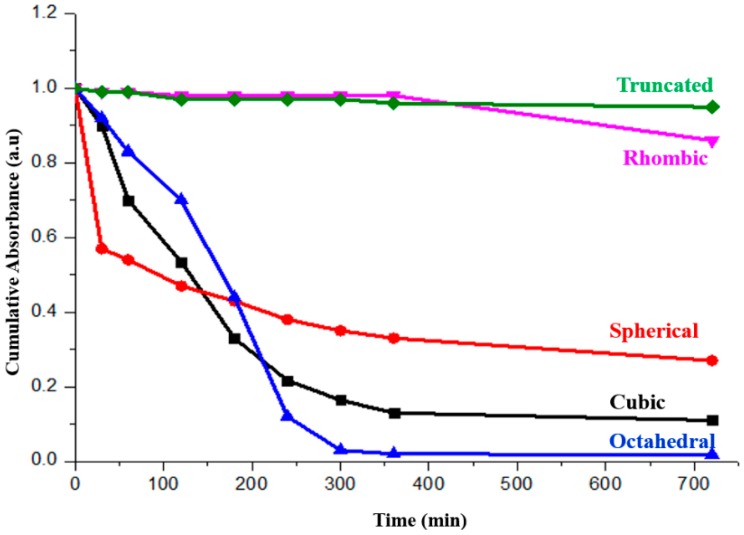
Adsorption performance of the Cu_2_O (spherical, cubic, octahedral, rhombic dodecahedral and truncated polyhedral) particles in 20 ppm methyl orange (MO) solution.

The adsorption performance of the Cu_2_O particles was evaluated using solution of MO, a negatively charged dye (Figure 8). It was observed that Cu_2_O particles with positive surface charges (spherical, cubic and octahedral) were able to adsorb MO due to the electrostatic force. The adsorption was almost complete after 300 min for the octahedral Cu_2_O. Both the rhombic dodecahedral and truncated Cu_2_O particles possess negative charges, and there was nearly no adsorption due to electrostatic repulsion. Based on the zeta potential results shown earlier (Table 2), it was observed that the octahedral sample was more positively charged, followed by the cubic and then the spherical structures. The sequence agrees well with the adsorption performance despite the difference in the specific area: although the spherical sample possessed a higher surface area, the adsorption was observed to be slower and no further adsorption could be seen after 360 min. Hence, it is proven that the surface charge critically affects the adsorption performance of the synthesized Cu_2_O particles.

**Figure 9:**
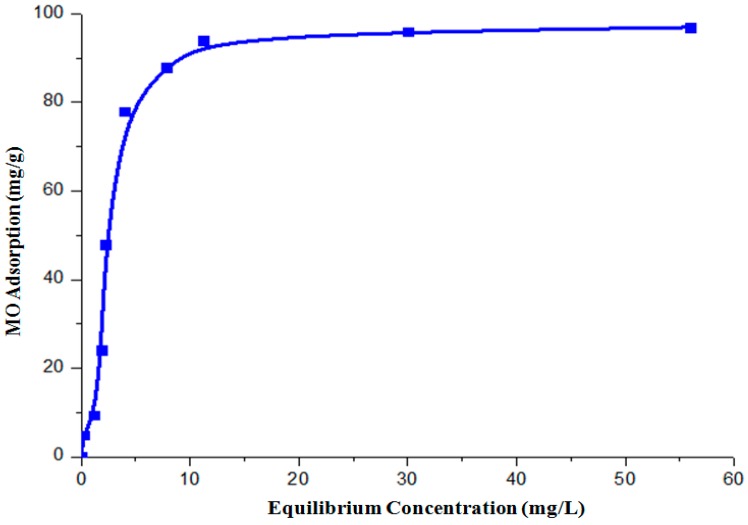
The adsorption isotherms of the octahedral shaped Cu_2_O.

As the adsorption performance of octahedral sample was evidently the best, it is of interest to further understand the adsorption mechanism and capacity of this material. Figure 9 presents the adsorption isotherm of the Cu_2_O octahedral shaped sample.

The curve is fitted into the well-known Freundlich (Equation (1)) and Langmuir (Equation (2)) models:

(1) $$\mathrm{Freundlich}:\;q\;=\;K_{f}C^{\frac{1}{n}}$$

(2) $$\mathrm{Langmuir}:\;q\;=\;\frac{q_{m}\mathrm{KC}}{1\;+\;\mathrm{KC}}\;$$

where q (mg/g) is the amount of adsorbed MO, C the concentration of MO at equilibrium, q_m_ (mg/g) the maximum adsorption capacity, K_f_ and n are Freundlich constants and K is the Langmuir constant. The amount of MO adsorbed onto the photocatalyst, q, is given by:

(3) $$q\;=\;\frac{{(C}_{o}-C_{f})V}{M}$$

where C_o_ and C_f_ represent the initial and final concentration of MO in the solution, V is the volume of MO solution (L) and M is the mass of photocatalyst added (g). The Freundlich and Langmuir models can be linearized as:

(4) $$\mathrm{Freundlich}:\;\mathrm{In}\;q_{e}\;=\;\mathrm{In}\;K_{F}\;+\;(\frac{1}{n})\;\mathrm{In}\;C_{e}$$

(5) $$\mathrm{Langmuir}\;:\;\frac{1}{q_{e}}=\frac{1}{q_{\max}K_{L}C_{e}}+\frac{1}{q_{\max}}$$

If the adsorption isotherm exhibits Langmuir behavior, it indicates monolayer adsorption. In contrast, a good fit into the Freundlich model indicates a heterogeneous surface binding. The results of fitting the isotherm curves to Freundlich and Langmuir models are summarized in Table 3. The fitting is clearly better for the Langmuir model. On the other hand, the R^2^ value is less than 0.8 when the data is fitted with the Freundlich model. This illustrates that the adsorption of MO by the octahedral Cu_2_O is governed by the monolayer adsorption. The MO adsorbed (q_m_) by the octahedral shaped Cu_2_O sample was found to be 96.42 mg/g (experimentally) and 66.0 mg/g (theoretically). The values are comparable with some reported adsorbents such as rice husk (40.58 mg/g) [22] and raw date pits (80.29 mg/g) [23]. 

### 2.4. The Photocatalytic Performance of Cu_2_O Particles

The photocatalytic activity of the as-prepared Cu_2_O samples was evaluated by degradation of MO under visible light irradiation. Photolysis test was also carried out as a reference. Water loss due to evaporation was calibrated when reporting the dye concentration.

**Figure 10:**
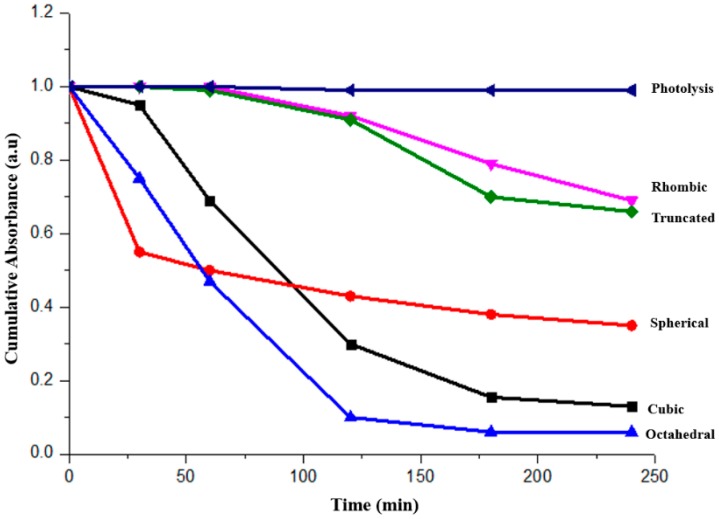
The photocatalytic performance of the faceted Cu_2_O (spherical, cubic, octahedral, rhombic dodecahedral and truncated polyhedral) particles under visible light irradiation.

Figure 10 shows the photocatalytic performance. When assessing the photocatalytic activity, reference has to be made with the adsorption experiment as shown in Figure 8. Through comparison, it is evident that the octahedral and cubic shaped Cu_2_O particles demonstrated substantial photocatalytic degradation of MO under visible light irradiation. The demonstrated good activity is attributed to the adsorption of the dye to the surface before the photo degradation occurs.

The rhombic dodecahedral and truncated Cu_2_O showed limited activity despite the fact that their negative surface charge repels the dye molecules. Huang et al. [24] have attributed the poor photocatalytic activity of the rhombic dodecahedral shaped Cu_2_O to the surface residues from the oleic acid used during the synthesis. However, as discussed earlier, in the current study, no FTIR peaks related to oleic acid could be found. Therefore, our current work confirms that the rhombic dodecahedral Cu_2_O indeed possesses some degree of photocatalytic activity.

**Figure 11:**
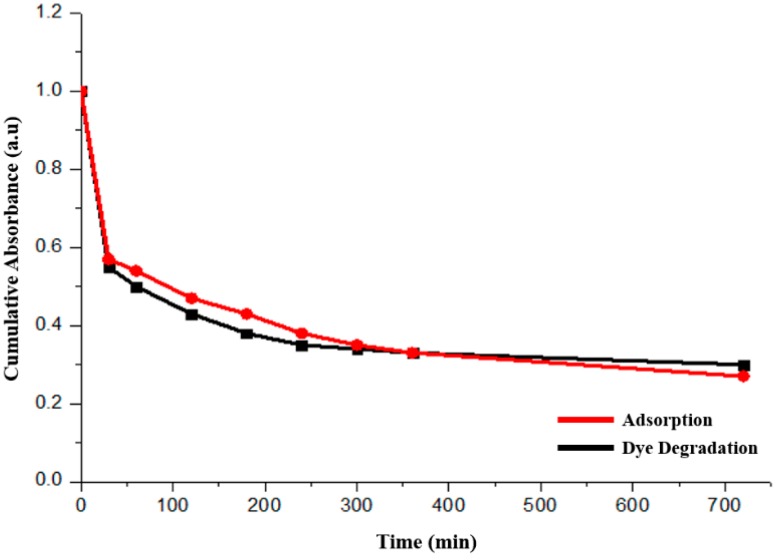
Comparison of adsorption (red line) and dye degradation (black line) performances of the spherical Cu_2_O particles.

No obvious dye degradation was observed for spherical particles once the adsorption curve is plotted together with the photocatalytic one, as shown in Figure 11. The poor crystallinity could be the main reason, which has resulted in the fast recombination of the photo-generated electron–hole pairs. 

### 2.5. Band Gap, Band Edge Positions and Proposed Photodegradation Mechanism

To determine the flatband potential, Mott–Schottky graph was obtained by measuring the apparent capacitance as a function of potential under depletion condition at the semiconductor–electrolyte junction based on:

(6) $$\frac{1}{{C_{sc}}^{2}}=\frac{2}{e\varepsilon \varepsilon_{0}N}\left(E-E_{fb}-\frac{kT}{e}\right)$$

where C_sc_ is the capacitance of the space charged region, e the electron charge (1.602 × 10^−19^ C), ε the diaelectric constant of the semiconductor, ε_0_ the permittivity of free space (8.85 × 10^−14^ F cm^−1^), N the donor density, E the applied potential, E_fb_ the flatband potential, k the Boltzmann constant (1.38 × 10^−23^ J K^−1^), and T the absolute temperature. Extrapolation of the linear plot to the applied potential axis leads to the value for E_fb_. Table 4 summaries the valence band potential position, the type of semiconductor, and the optical bandgap values that were determined by the optical absorption measurement.

**Figure 12:**
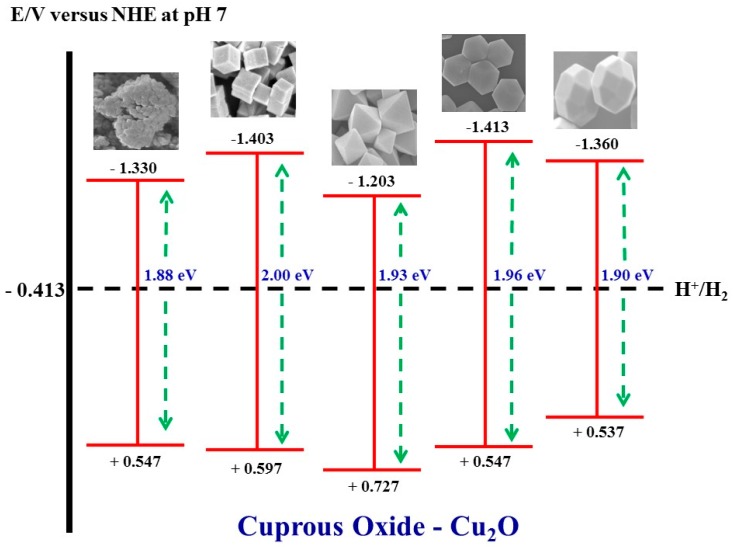
The band edge potential positions of the faceted Cu_2_O particles.

Figure 12 shows the band edge positions (with reference to NHE at pH ~7) and band gap energy of different Cu_2_O samples. The measured bandgap value only slightly varies from 1.9 to 2.0 eV, and the largest difference in the valence band position is about 0.19 eV. The difference in the band gap energy and band potential positions could be due to the size and the specific exposed facet(s) [25]. It was reported that the main oxidation species for MO degradation are ^•^OH radicals under our experimental pH condition [26]. In general, there are two routes that the ^•^OH radicals are generated, viz., through the photo-generated holes in the valence band or the photo-generated electrons in the conduction band. However, none of the particles are able to produce ^•^OH radicals directly from the photo-generated holes as the required semiconductor valence band potential is +1.58 eV for pH = 7 (+1.99 eV vs. NHE [27]), far more anodic than valence band potentials of all these Cu_2_O particles. Therefore, the mechanism of ^•^OH radical generation should be through the photogenerated electrons from the conduction band. The electrons first react with adsorbed oxygen to generate O_2_^−•^ and H_2_O_2_, which then react to form ^•^OH radicals [25].

In addition to the photocatalytic action discussed above, there is another possible dye degradation mechanism through the so-called photo-assisted degradation. Under such mechanism, the electrons generated by the dyes after light adsorption are injected to the conduction band of the photocatalyst. The electrons generated by the dye itself, rather than by the photocatalyst in a typical photocatalytic reaction, will cause chemical destruction of the dye molecules [28]. The LUMO (lowest unoccupied molecular orbital) energy of MO is around −1.636 eV with reference to NHE at ~pH 7 [29], so it is possible for the electrons to be injected from MO to the conduction band of Cu_2_O, leading to the photo-assisted degradation of MO itself. Because the energy gap between the LUMO and HOMO (highest occupied molecular orbital) of MO is about 2.35 eV [29], which is larger than the band gap of the Cu_2_O samples (1.9–2.0 eV), it becomes impossible to choose a light illumination that only excites the dye but not the photocatalyst—such experiment would be able to differentiate the photocatalytic action vs. the photo-assisted degradation. However, based on the fact that the spherical samples did not degrade MO, it is reasonable to deduce that the photo-assisted degradation is not likely to be a main contributing mechanism since it would not be affected by the charge recombination in the photocatalyst. In other words, if the photo-assisted degradation does exist, it should be manifested through the spherical particles.

### 2.6. Stability of the Synthesized Cu_2_O Particles

The stability of Cu_2_O is determined via the solar light illumination of intensity 100 mW/cm^2^ for 12 h when the particles were dispersed in MO solution. The samples were collected and analyzed using scanning electron microcopy (SEM), X-ray photoelectron microcopy (XPS) and FTIR. Comparison was made with 25% methanol (v/v) added to the solution to understand the source of the photocorrosion. 

**Figure 13:**
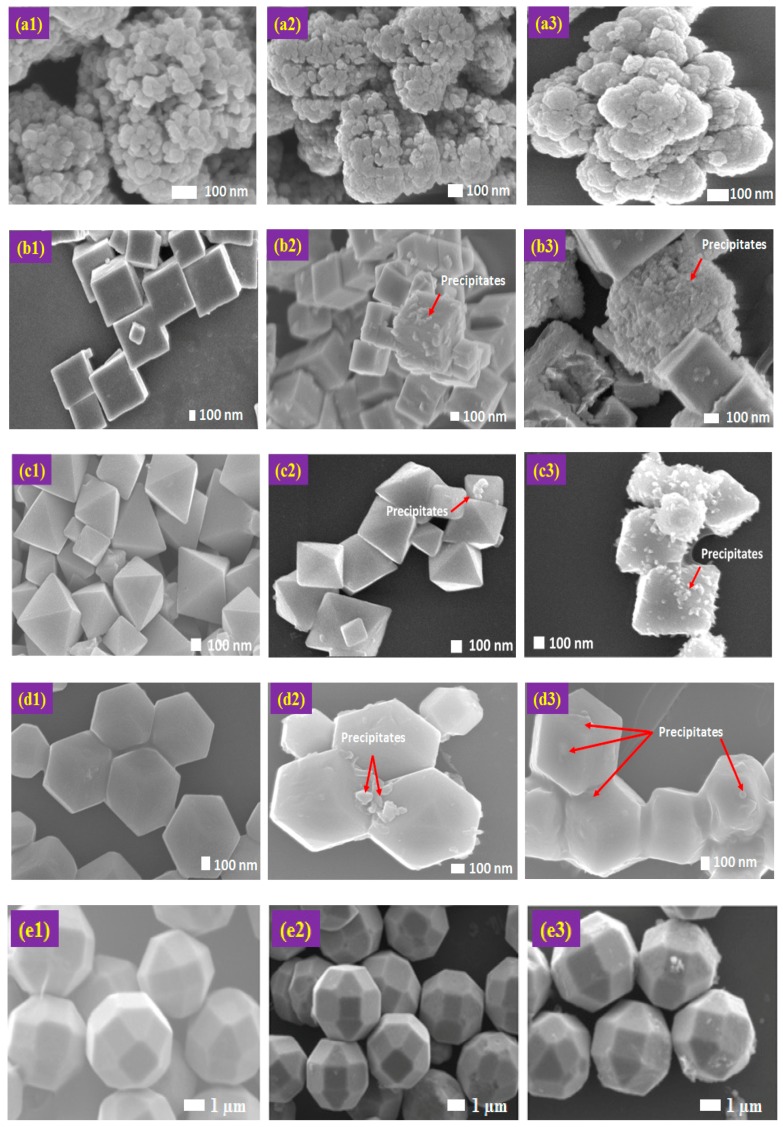
Column 1—FESEM images of as-synthesized Cu_2_O: (**a1**) spherical; (**b1**) cubic; (**c1**) octahedral; (**d1**) rhombic dodecahedral; and (**e1**) truncated polyhedral. Column 2—FESEM images of Cu_2_O in mixture of MO and methanol solution under solar light illumination for 12 h: (**a2**) spherical; (**b2**) cubic; (**c2**) octahedral; (**d2**) rhombic dodecahedral; and (**e2**) truncated polyhedral. Column 3—FESEM images of Cu_2_O in pure MO solution under solar light illumination for 12 h: (**a3**) spherical; (**b3**) cubic; (**c3**) octahedral; (**d3**) rhombic dodecahedral; and (**e3**) truncated polyhedral.

**Figure 14:**
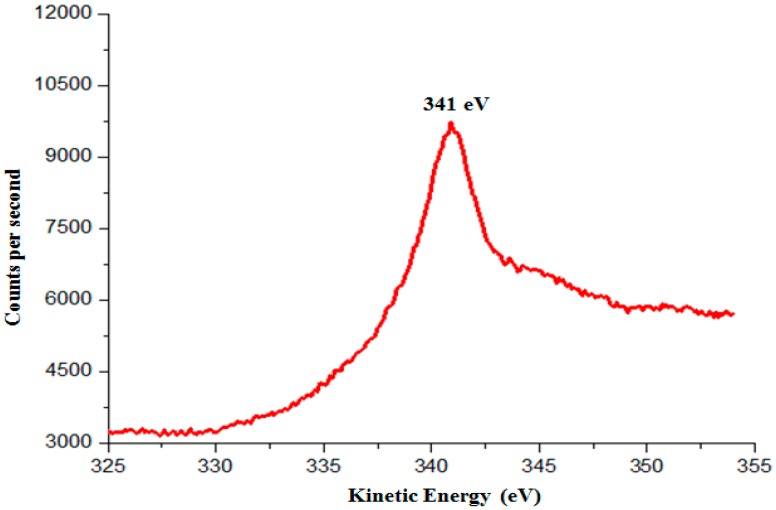
The kinetic energy (Auger LMM) of the photocorroded Cu_2_O particles.

Figure 13 compares the morphology of the faceted Cu_2_O particles at the as-synthesized state (column 1), after light illumination in the (MO + methanol) solution (column 2), and after light illumination in the MO solution (column 3). Comparing the first and the third columns, it is observed that the spherical shaped (Figure 14a) Cu_2_O particles do not seem to have suffered much photocorrosion. In relation to nearly zero photocatalytic activity as reported earlier, it strongly suggests that corrosion of the Cu_2_O is related to photo-generated species.

In the case of the cubic and octahedral shaped Cu_2_O particles (Figure 13b,c), small amount of precipitates could be observed on the surface. For the rhombic dodecahedral Cu_2_O particles (Figure 13d), the precipitates could be seen on the surface of the particles. On the other hand, the truncated polyhedral Cu_2_O (Figure 13e) particles suffered from minimum photocorrosion as only a very small amount of precipitates could be found on the surface.

The addition of methanol, a hole scavenger, in the MO solution has clearly alleviated the severity of photocorrosion (comparing columns 2 and 3). This suggests that the photocatalytically generated holes are responsible for the photocorrosion of Cu_2_O.

To determine the corrosion mechanism, we took the octahedral sample as a representative and exposed it under the solar light irradiation for an extended period of 120 h. After that, XPS and FTIR were employed in the analysis of the corrosion products on the particle surface. XRD was also explored but the intensity of the corrosion products was not strong enough for a firm identification.

**Figure 15:**
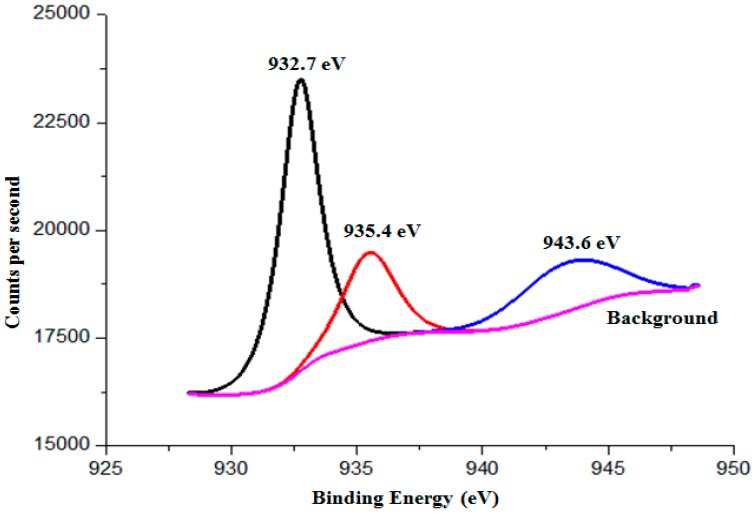
The binding energy of the photocorroded Cu_2_O particles.

Possible photocorrosion products of Cu_2_O include Cu, Cu(OH)_2_ and CuO. As the binding energies for Cu^0^ and Cu^1+^ are 932.67 eV and 932.6 eV, respectively [30], it is difficult to determine the phase of the photocorrosion products through Cu 2p_3/2_ scan. As such, XPS scan for the Auger peak of Cu LMM is needed to differentiate between Cu^0^ and Cu^1+^. The Auger LMM energy for Cu^0^ and Cu^1+^ lie in 334.95 eV and 336.80 eV respectively [31]. Figure 14 shows the Auger LMM spectra of octahedral shaped Cu_2_O particles which have Auger LMM energy of 341 eV. This Auger energy generated corresponds to the Auger energy of Cu^1+^. Figure 15 shows the Cu 2p_3/2_ XPS spectra of the Cu_2_O particles. The main peaks at 932.7 and 935.4 eV correspond to the binding energies of Cu_2_O and Cu(OH)_2_ respectively [31,32]. Lastly, the satellite peaks on the higher binding energy, 943.6 eV, indicates the presence of an unfilled Cu 3d shell and thus confirms the existence of Cu^2+^ on the sample surface.

**Figure 16:**
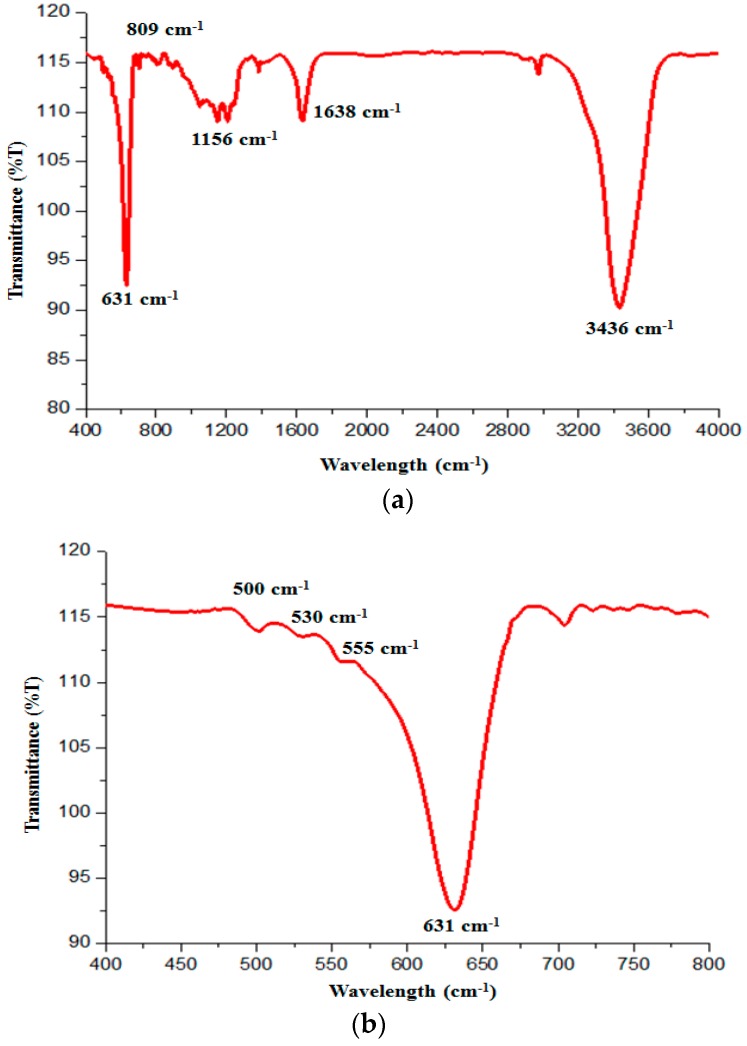
(**a**) FTIR spectrum of the photocorroded Cu_2_O particles; and (**b**) zoom-in FTIR showing the existence of CuO.

The XPS confirms that the top surface (4–8 nm) of the Cu_2_O particles contain Cu(OH)_2_ precipitates. To explore possible photocorrosion products beneath the top layer, FTIR measurement was used. As shown in Figure 16a, the broad bands centered at 3436 and 1638 cm^−1^ are attributed to the O–H stretching and bending modes of water [16,33]. The peaks located at 631, 809 and 1156 cm^−1^ are attributed to the stretching vibration of Cu–O in Cu_2_O.

When zooming into the details in the range of 400 to 800 cm^−1^, the adsorption band at 530 cm^−1^ associated with CuO is revealed (Figure 16b). The low intensity suggests that the amount of CuO is relatively small.

Based on the above analyses, the photocorrosion precipitates formed on the surface of Cu_2_O particles are mainly Cu(OH)_2_ and a small amount of CuO. This finding agrees with our early analysis that the photo-generated electrons are responsible for the MO degradation, while the holes are left to oxidize Cu^+^ to Cu^2+^ state. The presence of the photocorrosion products will hinder the photocatalytic activity of Cu_2_O as they block the light from reaching the Cu_2_O surface. Adding hole scavenger has alleviated the photocorrosion, and this observation also confirms that the holes are responsible for the photocorrosion of Cu_2_O.

## 3. Materials and Methods

### 3.1. Synthesis of Cu_2_O Particles

Copper(II) acetate monohydrate (C_4_H_6_CuO_4_·H_2_O, 99.0%) was purchased from Fluka (Singapore). Copper(II) sulfate pentahydrate (CuSO_4_·5H_2_O, 98.0%), l-ascorbic acid (C_6_H_8_O_6_, reagent grade), hydrazine hydrate (H_4_N_2_·xH_2_O, reagent grade 50–60%), oleic acid (C_18_H_34_O_2_, technical grade 90.0%), and d-(+)-Glucose (C_6_H_12_O_6_, 99.5%) were obtained from Sigma Aldrich (Singapore). Sodium hydroxide pellets (NaOH, 99.0%) were acquired from Schedelco (Singapore). Absolute ethanol (C_2_H_5_OH, 99.0%) was purchased from Merck (Singapore). Absolute methanol (CH_3_OH, analytical reagent grade) was purchased from Fisher Scientific (Singapore). Hexane (C_6_H_14_, reagent grade) was obtained from Riverbank Chemical Pte Ltd (Singapore). All chemicals were used as received without further purification.

#### 3.1.1. Spherical Cu_2_O Particles

Copper(II) acetate (Cu(OAc)_2_) (0.01 mol) was dissolved into 20 mL of deionized water under constant stirring at 500 rpm. Forty milliliters of 0.01 M NaOH was then added to the solution followed by 0.01 M of ascorbic acid (20 mL). After stirring for 30 min, the solution was centrifuged at 6000 rpm for 3 min and then washed with deionized water and ethanol solution. The powder collected was then dried in a vacuum oven at 60 °C for 6 h.

#### 3.1.2. Cubic Cu_2_O Particles

Copper(II) acetate (Cu(OAc)_2_) (0.01 mol) was dissolved in 20 mL of deionized water under constant stirring at 500 rpm. Forty milliliters NaOH solution (0.03 M) was then added, followed by 20 mL of 0.01 M ascorbic acid. After stirring for 30 min, the solution was centrifuged at 6000 rpm for 3 min followed by washing with deionized water and ethanol solution. The powder collected was then dried in a vacuum oven at 60 °C for 6 h.

#### 3.1.3. Octahedral Cu_2_O Particles

Copper(II) acetate (Cu(OAc)_2_) (0.01 mol) was dissolved in 20 mL of deionized water under constant stirring at 500 rpm. Forty milliliters NaOH solution (0.03 M) was then added to the solution followed by addition of 40 mL hydrazine hydrate solution (0.05 M). After 45 min of stirring, the solution was centrifuged at 6000 rpm for 3 min and washed with deionized water and ethanol solution. The powder collected was dried in a vacuum oven at 60 °C for 6 h.

#### 3.1.4. Rhombic Dodecahedral Cu_2_O Particles

Copper(II) sulfate (CuSO_4_·5H_2_O) (0.25 g) was dissolved in 40 mL deionized water. When the powder was fully dissolved, 5 mL oleic acid and 20 mL absolute ethanol were added to the solution. The solution was vigorously stirred at 700 rpm for 30 min before being heated at 90 °C. Twenty milliliters NaOH solution (0.08 M) was then added to the CuSO_4_ solution under constant stirring for 5 min at 90 °C. Afterwards, 3.42 g of glucose, which was dissolved in 30 mL of heated deionized water, was added to the solution. The final solution was left to stir at 90 °C for 45 min. After that, the solution was centrifuged and washed with hexane (10 times) and ethanol (3 times). The powder collected was then dried in a vacuum oven at 60 °C for 6 h. 

#### 3.1.5. Truncated Polyhedral Cu_2_O Particles

Copper(II) acetate (Cu(OAc)_2_) (0.015 mol) was dissolved in 40 mL deionized water at 70 °C. After continuous stirring at 500 rpm for 5 min, 10 mL of 9.0 M NaOH and 0.6 g glucose were added into the solution. The solution continued to be stirred at 70 °C for about 60 min before being cooled down to room temperature. The powder was washed and centrifuged with ethanol (6 times) and deionized water (3 times). Finally, the powder was dried in a vacuum oven at 60 °C for 6 h.

### 3.2. Materials Characterization

Crystal structure was identified by X-ray diffraction (XRD) using a Shimadzu LabX-6000 diffractometer (Shimadzu Corporation, Tokyo, Japan) with Cu Kα radiation (λ = 1.54178 Å). A step size of 0.02° over 2θ ranging from 10° to 80° was used with a scanning rate at 2.33° per minute. The accelerating voltage and emission current were 40 kV and 30 mA, respectively.

The morphology of the samples was examined by field emission scanning electron microscopy (FESEM, JEOL JSM-7600F, JEOL Ltd., Tokyo, Japan) and transmission electron microscopy (TEM, JEOL JEM-2010, JEOL Ltd., Tokyo, Japan). The specific surface areas were evaluated using a Micromeritics ASAP 2020 (Micromeritics Instrument Corporation, Norcross, GA, USA) surface analyzer based on the BET theory. The samples were outgassed under vacuum and heated to 100 °C before the test.

The surface chemical analysis was carried out by X-ray photoelectron spectroscopy (XPS) using a VG ESCALab 220i-XL system (Thermo Scientific, Waltham, MA, USA). Mg Kα X-ray (hν = 1253.6 eV) from twin anode X-ray gun was employed using a large area lens mode for analysis with photoelectron takeoff angle of 90° with respect to surface plane. The maximum analysis depth is in the range of 4–8 nm. Survey spectra were acquired for elemental identification while high-resolution spectra were acquired for chemical state identification and surface composition calculation. For chemical state analysis, a spectral deconvolution was performed by a curve-fitting procedure based on Lorentzians broadened by Gaussian using the manufacturer’s standard software. The error of binding energy is estimated to be within 0.2 eV.

FTIR was carried out in a Perkin Elmer Instruments Spectrum GX FTIR spectrometer (PerkinElmer, Waltham, MA, USA). Synthesized particles were mixed with standard KBr particles and pressed into thin pellets. The spectral range was 400–4000 cm^−1^ and a total of 40 scans were recorded at a resolution of 4 cm^−1^ averaging each spectrum. 

Zeta potential was measured by a Mavern Nanosizer system (Malvern Instruments Ltd, Malvern, UK). Particles (5–10 mg) were dispersed in deionized water (~pH 7) and sonicated for 5 min. The equilibrium time was set at 120 s and each sample was run for 10 times to obtain the average value of the surface charge. 

Optical absorption of bulk powders was measured on a Perkin Elmer Lambda 900 UV-Visible spectrometer (PerkinElmer, Waltham, MA, USA) in the diffuse reflectance spectroscopy mode over the spectra ranging from 250 to 800 nm. The optical diffuse reflectance spectrum of Cu_2_O was measured on a Shimadzu 2550 UV-vis-NIR spectrometer (Shimadzu Corporation, Tokyo, Japan) using BaSO_4_ as a reference standard. The bandgap of Cu_2_O was calculated using the Kubelka–Munk function. 

The flat band potentials were measured by impedance spectroscopy based on the Mott–Schottky plots. To prepare for the test samples, 15 mg of the as-synthesized Cu_2_O powder was sonicated in 1 mL of ethanol to obtain a homogeneous mixture. The Cu_2_O suspension was then drop-casted on a conductive fluorine-tin oxide (FTO) glass substrate with adhesive tapes acting as spacers attached on the four edges. The substrate was then dried at 80 °C, and the adhesive tape attached on the top side of the substrate was removed. Electrical contact was formed by first applying silver paint on the top uncoated area of FTO, and then sticking a conductive copper tape onto the dried silver paint. Three electrodes were used for the impedance measurements which include the working electrode (the Cu_2_O film), a Pt counter electrode, and a reference electrode (Ag/AgCl, saturated in KCl). A 0.1 M Na_2_SO_4_ solution was used as the electrolyte. The measurements were carried out by a Gamry electrochemical impedance spectrometer, and the potential was systemically varied between +0 V and +2.0 V with frequency of 50 Hz.

### 3.3. Photocatalytic and Adsorption Experiments

Methyl orange (MO) was chosen as a representative dye to test the photocatalytic degradation activity and the adsorption capacity of the prepared Cu_2_O samples. Solution of the dye was prepared by dissolving the dye in deionized water at 20 ppm concentration, and the solution pH was around 6.7. One hundred mg of the powder samples were dispersed in 50 mL MO solution for the dye degradation test under visible light irradiation. A 100 mL glass beaker, wrapped with aluminum foil on its side wall, was used as the reactor with light shone from the top. The irradiation source comes from a solar simulator equipped with a 300 W Xe-lamp (HAL-320, Asahi Spectra Co., Ltd., Kita-ku, Japan). Super cold filter (YSC0750) was used to provide visible light ranging from 420 to 700 nm. The light intensity was around 50 mW/cm^2^. The amount of photocatalyst used was chosen through a few trial runs based on their degradation speed.

An adsorption isotherm test was carried out in the dark to prevent the potential photocatalytic degradation of MO under light. The equilibrium adsorption isotherm was determined using various concentrations of MO (10, 20, 30, 50 and 80 ppm). For each test, 100 mg of adsorbent was added to 50 mL MO solution. After 48 h, the equilibrium concentration was measured.

To determine the adsorption capacity, 100 mg of synthesized powder was added to 50 mL MO solution of various concentrations (10, 20, 30, 50 and 80 ppm). Stirring was applied throughout the duration of the test (1440 min) at a speed of 400 rpm in dark. At different intervals, the sample was collected, centrifuged and measured using the UV-visible spectrometer to determine the dye concentration.

### 3.4. Photocatalytic Stability Study

Two separate tests were carried out to measure the photocatalytic stability of Cu_2_O particles and to determine the possible root cause of the instability. In the first experiment, 50 mg powder was dispersed in 50 mL MO solution with 10 ppm concentration. For the second experiment, 50 mg of the powder was dispersed in 40 mL of MO solution (10 ppm) mixed with 10 mL of absolute methanol (purity > 99%). Both were constantly stirred under a solar simulator equipped with a 300 W Xe-lamp (HAL-320, Asahi Spectra Co., Ltd., Kita-ku, Japan) for 12 h under the intensity of 100 mW/cm^2^. An AM 1.5 G filter (400 to 1100 nm) was used.

## 4. Conclusions

In this paper, we have studied the adsorption and photocatalytic performances of the various faceted Cu_2_O samples for MO removal from its solution. The adsorption capability of Cu_2_O particles was found to be mainly determined by the surface charge. The octahedral shaped Cu_2_O particles with exposed {111} facets performed the best due to its most positive surface charges. The adsorption of the octahedral shaped Cu_2_O was found to follow the Langmuir monolayer mechanism.

The spherical shaped Cu_2_O without clearly defined facets did not display photocatalytic activity. All the faceted samples showed different degree of photocatalytic activities. The octahedral shaped Cu_2_O particles with exposed {111} facets performed the best in photocatalytic degradation of MO under visible light. The photo-generated electrons are responsible for the degradation of MO solution, while the photo-generated holes attack Cu_2_O, causing photocorrosion. It was observed that the corrosion precipitates are mainly Cu(OH)_2_, together with a small amount of CuO. These photocorrosion products hinder the photocatalytic activity of Cu_2_O and thus shorten the service life of Cu_2_O particles. The addition of hole scavengers such as methanol has shown to have alleviated the corrosion attack on Cu_2_O.

## Figures and Tables

**Table 1:** Size and specific surface area of the synthesized Cu_2_O particles.

	Spherical	Cubic	Octahedral	Rhombic Dodecahedral	Truncated
Size (µm)	0.80 ± 0.15	0.75 ± 0.37	0.65 ± 0.08	0.80 ± 0.157	5.0 ± 0.37
Surface area (m^2^/g)	3.95	1.13	1.13	0.34	Not available

**Table 2:** Zeta potentials of the faceted Cu_2_O.

Zeta Potential (mV)	Spherical (+)	Cubic (+)	Octahedral (+)	Rhombic Dodecahedral (−)	Truncated (−)
Test 1	5.93	6.70	19.7	−15.3	−4.59
Test 2	5.76	7.63	20.9	−15.1	−2.99
Test 3	5.83	6.25	23.1	−15.3	−3.41
Test 4	4.92	4.51	31.8	−17.6	−2.68
Test 5	3.33	5.96	30.6	−18.9	−1.45
Test 6	5.79	5.63	31.5	−18.1	−1.75
Test 7	5.79	5.88	33.0	−18.3	−5.41
Test 8	5.53	5.27	31.9	−7.84	−3.93
Test 9	4.51	6.31	31.3	−7.84	−1.66
Test 10	3.33	5.16	32.9	−7.78	−3.24
**Range**	3.33 to 5.93	4.51 to 7.63	19.7 to 33.0	−7.68 to −18.9	−1.45 to −4.59

**Table 3:** Adsorption isotherm parameters fitted to Langmuir and Freundlich models.

	Langmuir				Freundlich		
**Adsorbent**	q_m_ (exp) (mg/g)	K_L_ (L/mg)	q_m1_ (mg/g)	R^2^	K_F_ (mg^1−n^L^−n^g^−1^)	n	R^2^
**Octahedral**	96.42	0.40	66	0.86	17.3	1.79	0.76

**Table 4:** The tabulated valence band position and optical bandgap for the Cu_2_O particles.

Particle	Valence Band Position vs. NHE (eV)	Type	Optical Bandgap (eV)
Spherical	+0.547	p-type	1.88
Cubic	+0.597	p-type	2.00
Octahedral	+0.727	p-type	1.93
Rhombic Dodecahedral	+0.547	p-type	1.96
Truncated Polyhedral	+0.537	p-type	1.90
